# Physical activity, dietary intake and quality of life during COVID-19 lockdown in patients awaiting transcatheter aortic valve implantation

**DOI:** 10.1007/s12471-021-01609-z

**Published:** 2021-08-09

**Authors:** D. van Erck, C. D. Dolman, M. Snaterse, M. Tieland, A. H. G. Driessen, P. J. M. Weijs, W. J. M. Scholte op Reimer, J. P. Henriques, J. D. Schoufour

**Affiliations:** 1 Department of Cardiology, Amsterdam University Medical Centers, location Academic Medical Center, University of Amsterdam, Amsterdam, The Netherlands; 2 Department of Cardiothoracic Surgery, Amsterdam University Medical Centers, location Academic Medical Center, University of Amsterdam, Amsterdam, The Netherlands; 3 Faculty Health, Center of Expertise Urban Vitality, Amsterdam University of Applied Sciences, Amsterdam, The Netherlands; 4 Faculty of Sports and Nutrition, Center of Expertise Urban Vitality, Amsterdam University of Applied Sciences, Amsterdam, The Netherlands

**Keywords:** Exercise, Transcatheter aortic valve implantation, COVID-19, Quality of life

## Abstract

**Background:**

The COVID-19 pandemic has led to a national lockdown in the Netherlands, which also affected transcatheter aortic valve implantation (TAVI) patients. The objective of the study was to describe physical activity, dietary intake and quality of life (QoL) in patients on the waiting list for TAVI pre-lockdown and during lockdown.

**Methods:**

Consecutive patients awaiting TAVI at the Amsterdam University Medical Centers, the Netherlands were included. Measurements were self-reported effect of lockdown, physical activity, dietary intake and QoL.

**Results:**

In total, 58 patients (median age 80, interquartile range (IQR) 76–84, 45% female) were observed pre-lockdown and 16 patients (median age 78, IQR 76–82, 25% female) during lockdown. Ten of the 16 patients during lockdown reported a decline in physical activity. However, we observed a median number of 5861 steps a day (IQR 4579–7074) pre-lockdown and 8404 steps a day (IQR 7653–10,829) during lockdown. Median daily protein intake was 69 g (IQR 59–82) pre-lockdown and 90 g (IQR 68–107) during lockdown. Self-rated health on a visual analogue scale was 63 points (IQR 51–74) pre-lockdown and 73 points (IQR 65–86) during lockdown.

**Conclusions:**

More than half of the patients during lockdown reported less physical activity, while we observed a higher number of steps a day, a similar dietary intake and a higher QoL. Therefore, patients on the TAVI waiting list appeared to be able to cope with the lockdown measures.

## What’s new?

- In this study, most patients waiting for transcatheter aortic valve implantation (TAVI) reported engaging in less physical activity but doing extra activities during the COVID-19 lockdown in the spring of 2020.
- Physical activity level was higher during the lockdown than before the lockdown.
- Dietary intake was similar during and before the lockdown.
- Quality of life was higher during the lockdown than before the lockdown.

## Introduction

The outbreak of coronavirus disease 2019 (COVID-19) has been declared a public health emergency and most countries have introduced measures to slow down the spread, for example a nationwide lockdown. Older adults on the waiting list for a cardiac intervention such as transcatheter aortic valve implantation (TAVI) can experience negative effects of the COVID-19 measures in two ways.

First, this patient group has a significant risk of becoming seriously ill if they were infected with severe acute respiratory syndrome coronavirus 2 (SARS-CoV-2), as they are generally older than 70 years and cardiac patients are at higher risk of SARS-CoV‑2 infection [1]. Due to their high-risk status, they were strongly advised to minimalise social contact, stay at home as much as possible and not to have visitors during the lockdown.

Second, hospitals were overwhelmed with COVID-19 patients, which caused regular care to be postponed. Patients on the waiting list for a cardiac intervention had to deal with their health problems longer, such as shortness of breath and tiredness, and may potentially have been at higher risk of death. Additionally, patients are increasingly inactive in the period before a cardiac intervention [2, 3]. The combination of long-term passive waiting for treatment, the persistence of health problems, and the lack of activities and social contact could lead to a severe decline in physical activity, dietary intake and quality of life (QoL). Insufficient physical activity and insufficient protein intake lead to less muscle mass, decreased physical functioning, less independency, early mortality and a further decline in QoL [4, 5].

Several studies have indicated that individuals experience a decrease in physical activity level, dietary intake and QoL due to lockdown measures [6–9]. However, studies measuring physical activity, dietary intake and QoL among TAVI patients during lockdown are currently lacking. Therefore, we aimed to describe the physical activity, dietary intake and QoL of patients on the waiting list for TAVI pre-lockdown and during lockdown.

## Methods

### Study design and population

All consecutive preoperative patients discussed in the Heart Team and accepted for a transfemoral TAVI at the Amsterdam University Medical Centers in Amsterdam, the Netherlands were asked to participate in this cross-sectional observational study. The study was approved by the hospital’s ethics committee and patients provided informed consent.

Pre-lockdown data were part of two cohort studies. The first study focused on dietary intake and data were collected from May 2019 until January 2020. The second study focused on physical activity and QoL, which started data collection in January 2020 but had to stop in March 2020 because of the COVID-19 pandemic. From 12 March through 1 July 2020, we continued data collection on all three outcome measures, albeit in a modified form, by amending our methods to the COVID-19 protective measures. This meant patient inclusion and interview-based questionnaires were carried out by telephone and an activity tracker was sent to patients by mail after disinfection.

### Data collection and processing

The patients who were included during lockdown were asked three multiple choice questions on perceived effects of the COVID-19 measures on their physical activity level and dietary intake.

Physical activity and sedentary time were objectively measured as time-stamped steps with the Stepwatch 4 (Modus Health LLC, Edmonds, WA, USA), which is a valid tool to determine physical activity in older adults with normal, slow or irregular gait speed [10]. The Stepwatch had to be worn for 7 consecutive days during every waking hour for at least 12 h per day. Data were visually checked for completeness and patients with more than 3 days of complete data were included in the analysis. Thresholds for moderate and vigorous intensity were 100 and 130 steps per minute, respectively [11].

Dietary intake was determined with a 3-consecutive day dietary record. To increase validity, a trained researcher contacted each participant afterwards to discuss and complete the record [12]. To minimise recall bias, the dietary record was filled in three days before the meeting with the trained researcher. Day type (week or weekend day) was not taken into consideration as the difference in dietary intake between week and weekend days is minimal in older patients [13]. The dietary intake was converted to nutrient intake with the Dutch National Food Composition Database of 2016 [14].

Health-related QoL was determined with the EuroQol 5 Dimensions, which includes self-rated health on a vertical visual analogue scale (VAS) [15].

### Statistical analysis

Data are presented as mean and standard deviation (SD) for normally distributed data or median and interquartile range (IQR) for non-normally distributed data. Differences between groups were determined with an independent-sample *t*-test or Mann-Whitney U test depending on normality and with a chi-square or Fisher’s exact test for categorical data. Fischer’s exact test was used when the expected count was < 5. A *p*-value < 0.05 was considered statistically significant. Analyses were performed in RStudio (v3.6.0).

## Results

**Fig. 1:**
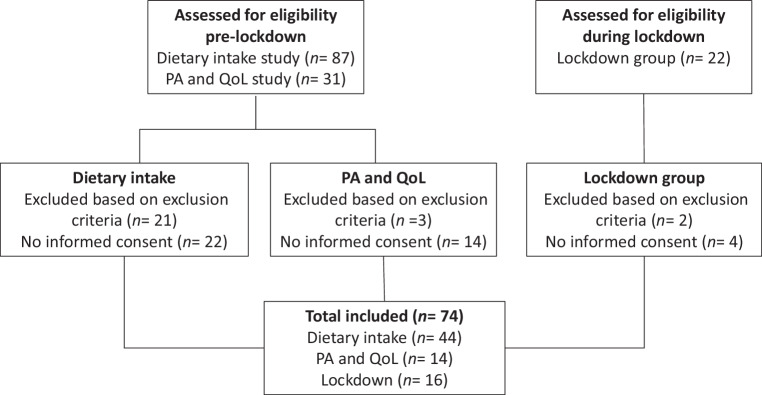
Study flow diagram. *PA* physical activity, *QoL* quality of life

The patient selection process is shown in Fig. 1. In total, 140 patients were assessed; 40 patients did not give consent. The pre-lockdown group consisted of 58 patients and the lockdown group of 16 patients. Median age was 80 years (IQR 76–84) in the pre-lockdown group and 78 years (IQR 76–82) in the lockdown group (Tab. 1). In the pre-lockdown group, 45% of the patients was female versus 25% of the patients in the lockdown group. Average waiting time between acceptation for TAVI and treatment was comparable: 50.8 days (SD 27.8) pre-lockdown and 55.8 (SD 40.4) during lockdown. None of the patients in either group died before TAVI.

**Table 1:** Characteristics of patients on waiting list for transcatheter aortic valve implantation, pre-lockdown and during lockdown

Characteristic	Pre-lockdown group (dietary intake) (*n* = 44)	Pre-lockdown group (PA and QoL) (*n* = 14)	Lockdown group (*n* = 16)	*P*-value
Age, years	80 (76–83)	81 (79–85)	78 (76–82)	0.406
Female sex	21 (47.7)	5 (35.7)	4 (25.0)	0.262
COPD	7 (15.9)	2 (14.3)	1 (6.2)	0.623
DM	16 (36.4)	4 (28.6)	3 (18.8)	0.417
Hypertension	31 (70.5)	9 (64.3)	11 (68.8)	0.910
*NYHA class*				0.020
I	3 (6.8)	3 (21.4)	3 (18.8)	
II	16 (36.4)	9 (64.3)	11 (68.8)	
III	20 (45.5)	2 (14.3)	2 (12.5)	
IV	5 (11.4)	0	0	
*LVEF*				0.238
Good (>50%)	34 (77.3)	10 (76.9)	11 (68.8)	
Moderate (31–50%)	10 (22.7)	2 (15.4)	5 (31.2)	
Poor (21–30%)	0	1 (7.7)	0	
Very poor (≤20%)	0	0	0	
AVA	0.84 ± 0.16	0.79 ± 0.15	0.84 ± 0.15	0.578
Maximum gradient	67.70 ± 17.22	65.07 ± 20.20	65.41 ± 16.16	0.840
STS score	2.01 (1.61–2.59)	1.80 (1.18–2.60)	1.38 (1.14–1.71)	0.073
*Edmonton frailty score*ª				0.471
No frailty	31 (70.5)	11 (78.6)	14 (93.3)	
Mild frailty	10 (22.7)	2 (14.3)	1 (6.7)	
Moderate frailty	3 (6.8)	1 (7.1)	0	
Severe frailty	0	0	0	

Data are median (interquartile range), *n* (%) or mean ± standard deviation

*PA* physical activity, *QoL* quality of life, *COPD* chronic obstructive pulmonary disease, *DM* diabetes mellitus, *NYHA* New York Heart Association, *LVEF* left ventricular ejection fraction, *AVA* aortic valve area, *STS* Society of Thoracic Surgeons

^a^For one patient in the lockdown group, there was no frailty score available

The comprehensive results are presented in Tab. 2. In brief, 10 of the 16 patients in the lockdown group reported a decline in physical activity due to the lockdown measures and 9 did extra home-based activities. One patient reported a change in dietary intake.

**Table 2:** Physical activity, dietary intake and quality of life in patients on waiting list for transcatheter aortic valve implantation, pre-lockdown and during lockdown

Variable	Pre-lockdown group	Lockdown group	*P*-value
*Self-reported change in physical activity*		*(n* *=* *16)*	
More		1 (6)	
Similar		5 (31)	
Somewhat less		8 (50)	
Much less		2 (13)	
Extra home-based activities		9 (56)	
Self-reported change in dietary intake		1 (6)	
*Physical activity*^*a*^	*(n* *=* *13)*	*(n* *=* *13)*	
Number of steps/day	5861 (4579–7074)	8404 (7653–10,829)	0.02
*Intensity, min/week*			
Light intensity	1698 ± 519	2047 ± 437	0.08
Moderate intensity	14 (4–70)	69 (0–145)	0.47
Vigorous intensity	0	0	0.25
Time out of bed, hours/day	14.0 (13.1–14.5)	14.7 (14.1–15.3)	0.05
Sedentary time, hours/day	9.57 ± 0.99	9.45 ± 1.29	0.79
*Dietary intake*^*b*^	*(n* *=* *44)*	*(n* *=* *12)*	
Energy intake, Kcal/day	1582 (1436–1917)	1813 (1466–2400)	0.23
Protein intake, gram/day	69 (59–82)	90 (68–107)	0.09
Protein intake, gram/kg per day	0.93 (0.75–1.09)	1.09 (0.84–1.30)	0.12
Carbohydrate intake, gram/day	183 (146–224)	201 (164–243)	0.23
Fat intake, gram/day	60 (49–72)	57 (49–84)	0.90
*Quality of life*^*c*^	*(n* *=* *14)*	*(n* *=* *16)*	
Health status^d^	63 (51–74)	73 (65–86)	0.05
*Mobility*			> 0.99
No problems	10 (71)	11 (69)	
Some problems	4 (29)	5 (31)	
Severe problems	0	0	
*Self-care*			0.47
No problems	13 (93)	16 (100)	
Some problem	1 (7)	0	
Severe problems	0	0	
*ADL*			
No problems	11 (79)	15 (94)	0.32
Some problems	3 (21)	1 (6)	
Severe problems	0	0	
*Pain*			> 0.99
No pain	10 (71)	12 (75)	
Some pain	4 (29)	4 (25)	
Severe pain	0	0	
*Depressive feelings*			0.85
No depressive feelings	8 (57)	10 (63)	
Some depressive feelings	5 (36)	6 (37)	
Severe depressive feelings	1 (7)	0	
*Memory*			> 0.99
No problems	11 (79)	12 (75)	
Some problem	3 (21)	4 (25)	
Severe problems	0	0	

Data are *n* (%), median (interquartile range) or mean ± standard deviation

*ADL* Activities of Daily Living

^a^Physical activity assessed by Stepwatch

^b^Dietary intake assessed by dietary record

^c^Quality of life assessed by EuroQol 5 Dimensions

^d^Health status assessed by visual analogue scale

The median number of steps a day was 5861 (IQR 4579–7074) pre-lockdown versus 8404 (IQR 7653–10,829) during lockdown. In both groups combined, 2 of the 26 patients had a sedentary time of less than 8 h. The median energy intake was 1582 kcal/day (IQR 1436–1917) in the pre-lockdown group and 1813 kcal/day (IQR 1466–2400) in the lockdown group, whereas the median daily protein intake was 69 g (IQR 59–82) and 90 g (IQR 68–107), respectively. Ten of the 56 patients for whom data on the protein intake were available, met the recommended protein intake of 1.2 g/kg per day. Lastly, the median VAS score (QoL) was 63 (IQR 51–74) pre-lockdown and 73 (IQR 65–86) during lockdown.

## Discussion

We observed that older cardiac patients awaiting TAVI who were included in our study during lockdown walked a higher number of steps, had a similar dietary intake and reported a higher QoL compared with those included pre-lockdown. Furthermore, most patients were sedentary and did not meet the recommended levels for physical activity and protein intake.

More than half of the patients experienced self-reported decline in physical activity due to the COVID-19 measures. However, most of the patients who reported less physical activity also said they undertook extra activities. During the lockdown, much media attention was directed to physical activity. Combined with increased health awareness, this caused more interest in physical activity during the lockdown [16]. This increased awareness could have motivated patients to become more active and could therefore have resulted in their being more physically active [17].

In addition, seasonality could have affected the results. It is well known that during the spring (the studied lockdown took place in spring), people become more active when the days are longer and the weather is better. However, this effect is often less persistent in adults older than 75 years [18].

Although we observed a higher number of steps during lockdown, it is evident that both groups did not meet the recommended amount of physical activity and were sedentary. The intensity of most activity was light and fell far below the recommended 150 min at moderate or 75 min at vigorous intensity [19]. All but two patients had a total sedentary time of more than 8 h, indicating a sedentary lifestyle, which is associated with early mortality [20]. Therefore, sedentary time needs to be decreased and activity at a moderate intensity needs to be increased in preoperative TAVI patients.

For dietary intake, only one patient reported a change during the lockdown. This is consistent with our results that showed no differences in energy and macronutrient intake. In most of the patients in both groups, protein intake was below the recommended intake of 1.2 g/kg, while protein intake is very important to retain muscle mass and muscle function [21]. Therefore, protein intake in this patient group needs to be increased.

The VAS score for health status measured with the EuroQol 5 Dimensions was 10 points higher in the lockdown group than in the pre-lockdown group. There is an association between frailty, physical activity and QoL scores [22, 23]. In the lockdown group, 1 of the 16 patients was mildly frail (7%), while 2 out of 14 patients in the pre-lockdown group were mildly frail and 1 patient was moderately frail (21%). This could have resulted in a better health status in the lockdown group. Another explanation for the better quality of life could be the higher level of physical activity in the lockdown group [22]. A similar study in France and Switzerland showed that adults with increased activity during lockdown reported a higher physical health status [24].

### Study limitations

One limitation of the study is possible selection bias. However, during the lockdown, all referred patients were treated similarly to the normal situation, and acceptation for TAVI was independent of frailty or severity of symptoms. Therefore, we do not expect a selection bias based on the procedures for TAVI. This is supported by a lack of major differences in the baseline characteristics.

Further limitations are the small sample size, possible seasonal effects and the inability to perform repeated measures. Based on the sample size, no hard conclusions can be drawn and we are only able to describe the data. In addition, a substantial part of the patients did not provide informed consent. As they were frailer, our findings cannot be generalised to the most frail patients awaiting TAVI [25].

Despite these limitations, the study offers a valuable first insight into the effects of lockdown measures on older cardiac patients.

## Conclusion

This is the first study to measure physical activity, dietary intake and QoL in patients on the TAVI waiting list before and during a nationwide COVID-19 lockdown. Both groups had a sedentary lifestyle and insufficient protein intake. More than half of the patients reported a decline in physical activity level during lockdown, while a higher number of steps was observed. There were no meaningful differences in sedentary time and dietary intake, whereas QoL was higher during lockdown. These results offer a first insight into the effect of lockdown measures on patients awaiting TAVI. Since we observed no clear decline in physical activity, dietary intake and quality of life, older cardiac patients seem to be able to cope with the lockdown measures.
